# Artificial intelligence assisted patient blood and urine droplet pattern analysis for non-invasive and accurate diagnosis of bladder cancer

**DOI:** 10.1038/s41598-024-52728-7

**Published:** 2024-01-30

**Authors:** Ramiz Demir, Soner Koc, Deniz Gulfem Ozturk, Sukriye Bilir, Halil İbrahim Ozata, Rhodri Williams, John Christy, Yunus Akkoc, İlker Tinay, Cigdem Gunduz-Demir, Devrim Gozuacik

**Affiliations:** 1 Koç University Research Center for Translational Medicine (KUTTAM), Istanbul, Turkey; 2 Department of Computer Engineering, Koç University, Istanbul, Turkey; 3 SUNUM Nanotechnology Research and Application Center, Istanbul, Turkey; 4 Department of Surgery, Koç University, Istanbul, Turkey; 5 School of Engineering, University of Edinburgh, Edinburgh, UK; 6 Anadolu Medical Center, Gebze, Kocaeli Turkey; 7 KUIS AI Center, Koç University, Istanbul, Turkey; 8 School of Medicine, Koç University, Istanbul, Turkey

**Keywords:** Cancer, Machine learning

## Abstract

Bladder cancer is one of the most common cancer types in the urinary system. Yet, current bladder cancer diagnosis and follow-up techniques are time-consuming, expensive, and invasive. In the clinical practice, the gold standard for diagnosis remains invasive biopsy followed by histopathological analysis. In recent years, costly diagnostic tests involving the use of bladder cancer biomarkers have been developed, however these tests have high false-positive and false-negative rates limiting their reliability. Hence, there is an urgent need for the development of cost-effective, and non-invasive novel diagnosis methods. To address this gap, here we propose a quick, cheap, and reliable diagnostic method. Our approach relies on an artificial intelligence (AI) model to analyze droplet patterns of blood and urine samples obtained from patients and comparing them to cancer-free control subjects. The AI-assisted model in this study uses a deep neural network, a ResNet network, pre-trained on ImageNet datasets. Recognition and classification of complex patterns formed by dried urine or blood droplets under different conditions resulted in cancer diagnosis with a high specificity and sensitivity. Our approach can be systematically applied across droplets, enabling comparisons to reveal shared spatial behaviors and underlying morphological patterns. Our results support the fact that AI-based models have a great potential for non-invasive and accurate diagnosis of malignancies, including bladder cancer.

## Introduction

Bladder cancer (BCa), or urothelial carcinoma, is a common malignancy of the urinary tract. More than half a million new cases and hundreds of thousands of deaths are recorded globally every year^1^. BCa is four times more common in men than in women^2^. There are many risk factors predisposing to this cancer type, including tobacco smoking, infections, and exposure to various chemicals^3,4^.

BCa generally originates from the epithelial layer, the urothelium, which covers the inner surface of the bladder. According to invasiveness into the detrusor muscle, the muscularis propria of the bladder, BCa is classified as muscle-invasive bladder cancer (MIBC) and non-muscle invasive bladder cancer (NMIBC)^5^. Detailed classifications consider localization of the cancer to different layers of the bladder wall and further spread: Tumors at the carcinoma in situ (Cis or Tis) stage are flat and confined to the mucosa. pTa and T1 indicates confinement to mucosa and spread to the lamina propria (submucosa), respectively. pT2a and T2b denotes superficial and deep muscle invasion. pT3 tumors reach beyond the muscularis propria into the perivesical fat, and pT4 tumors invade adjacent organs and/or anatomical structures^6^. Unfortunately, approximately 20% of newly diagnosed patients present to the clinic as muscle invasive or metastatic cancer^7^. Due to high recurrence rates after treatment, even patients with non-aggressive disease undergo frequent cystoscopic examinations, which is a costly and invasive technique with possible complications.

At least 30 different molecules with BCa biomarker potential have been identified so far, but only a few of these markers have been approved for clinical use^5,8^. Tests that are currently used in the clinic and exploit markers include urine cytology, fluorescence in situ hybridization (FISH), Nuclear Matrix Protein (NMP-22) detection, BTA stat, BTA TRAK, ImmunoCyt/uCyt+, CertNDx, CxBladder tests^9,10^. High false positive and false negative rates of many of these tests limit the reliability of these diagnostic methods. Moreover, most of these methods are expensive. Hence, there is an urgent need for the development of more sensitive, specific, reliable, and cost-effective tests for the diagnosis of this cancer type.

Medical data can be obtained in a variety of forms and complexity, including clinical data, radiology images, pathology results^11^ electronical health records^12^, data from wearable sensors^13^, and more recently in the form of omics data^14^. Combination and interpretation of bulky data produced in healthcare systems sets the need for new systematic perspectives benefiting from advances in AI-based analysis methods. Consequently, artificial intelligence-(AI-)assisted analysis methods have recently emerged as promising tools for diagnosis of diseases, such as Alzheimer’s disease, cancer, diabetes, cardiovascular diseases, and stroke^15^.

Machine learning is a branch of AI in which computers leverage data to learn and perform a given task rather than being explicitly programmed with a predetermined set of rules^16^. Machine learning and deep learning for detection of cancer and therapy evaluation at a single-cell level were used in cell and cancer biology field. For instance, based on phenomic analyses, machine learning-assisted method was used to determine defects during embryogenesis as well as discriminate non-tumor and tumor cells in different cancer model^17–19^. Current AI-based approaches have also been tested by independent research groups for bladder cancer diagnosis, staging and grading of tumors, as well as for predicting response to chemotherapy, recurrence, and overall survival^20^. In these studies, imaging, cytology or histopathology data from BCa patients were used and convolutional neural networks (CNNs) were used as the most common AI model to classify the medical images^21^.

Blood and urine samples are among the most prevalent biological specimens used in the clinics for routine biochemical and cellular analysis. They are easily obtained from patients and their analysis reveals information relevant to patient healthcare. The health status of patients affects the composition, chemical properties, as well as physical and rheological properties of blood and urine^22,23^. Properties of these biological samples may differ in BCa patients compared to controls. In addition to passage into blood circulation via tumor vascularization, tumor-derived cells and secretions may be released into the urine^24^. Indeed secretion or release of abnormally high levels or forms of specific proteins might positively correlate with BCa^24^.

Changes in blood fluidity are determined by factors such as plasma viscosity, clotting, erythrocyte aggregation propensity, red blood cell deformability, adhesion properties of platelets, and leukocytes. Moreover, changes in the composition and concentration of plasma components, including various proteins and hormones, vesicles, lipids, nucleic acids, inorganic electrolytes, glucose, and other elements, may affect blood composition and properties. On the other hand, urine contains not only electrolytes and other chemical molecules, but it may also contain cells, such as epithelial cells, erythrocytes, and lymphocytes, as well as sugars, proteins, lipids, and nucleic acids^25^. As expected from its complex organic and inorganic composition, evaporation of deposited drops of blood and urine samples may lead to formation of elaborate motifs and patterns^26^. During the evaporation process, distribution of the contents through evaporation dynamics results in the formation of characteristic patterns forming various shapes, motifs, shadows, and cracks^23,26^. Therefore, an AI-based analysis of complex patterns formed by dried blood or urine samples from patients suffering from cancer, and their comparison with samples from healthy volunteers might be used as a potential cancer diagnosis method.

In this study, droplet pattern analysis of evaporated deposits was performed on samples derived from BCa patients and compared to that of samples from healthy control subjects. Our proposed AI-assisted solution (a ResNet-18 network pre-trained on the ImageNet dataset) was systematically applied across blood and urine droplets^27^, enabling comparisons to reveal potentially shared spatial behaviors and underlying morphological patterns, which may precisely differentiate cancerous samples from controls. Thus, based on this approach, the identification of BCa patients was investigated, with the sensitivity and specificity of this method assessed statistically.

## Results

### Patients and control groups

A total of 130 human subjects with BCa diagnosis (110 male and 20 female) were included in the study. The control cohort group was composed of 64 volunteers (36 male and 28 female) who had no BCa diagnosis in their lifetime. Clinical and pathological characteristics and tumor classifications of the cohorts were summarized in Table 1. The median age of controls and BCa patients were calculated as 53 ± 16 and 66 ± 12, respectively. All tumors were diagnosed as urothelial cell carcinoma (UCC). The patient cohort was composed of primary (96 cases) or recurrent BCa cases (34 cases). According to invasiveness, patients were categorized as muscle non-invasive (NMIBC, 118 cases) or muscle invasive (MIBC, 12 cases). Tumor grades were also documented. Tumors were classified as low grade (61 cases) or high grade (68 cases) in the cohort. Tumor grades were determined as Cis (2 cases), pTa (67 cases), pT1 (49 cases), or pT2 (12 cases). Detailed information on patients and control cohorts was added as Supplementary Tables S1 and S2, respectively.

**Table 1:** Clinicopathological distribution of control individuals and bladder cancer patients.

	Classification	Sample number and gender (Male:Female)	Min. age (Years)	Max. age (Years)	Median age (Years)	Std. deviation ( ±)
Samples	Control	64 (36:28)	20	83	53	16
	Patient	130 (110:20)	23	89	66	12
Origin of tumor	Primary	96 (82:14)	23	89	66	12
	Recurrence	34 (28:6)	40	88	63	12
Invasiveness	NMIBC	118 (100:18)	23	89	66	12
	MIBC	12 (10:2)	49	86	67	12
Grade	Low grade	61 (53:8)	23	86	63	12
	High grade	68 (56:12)	37	89	68	11
Stage	Cis	2 (2:0)				
	pTa	67 (59:8)	23	88	63	12
	pT1	49 (39:10)	37	89	68	11
	pT2	12 (10:2)	49	86	67	12

### Imaging of blood droplets

**Figure 1:**
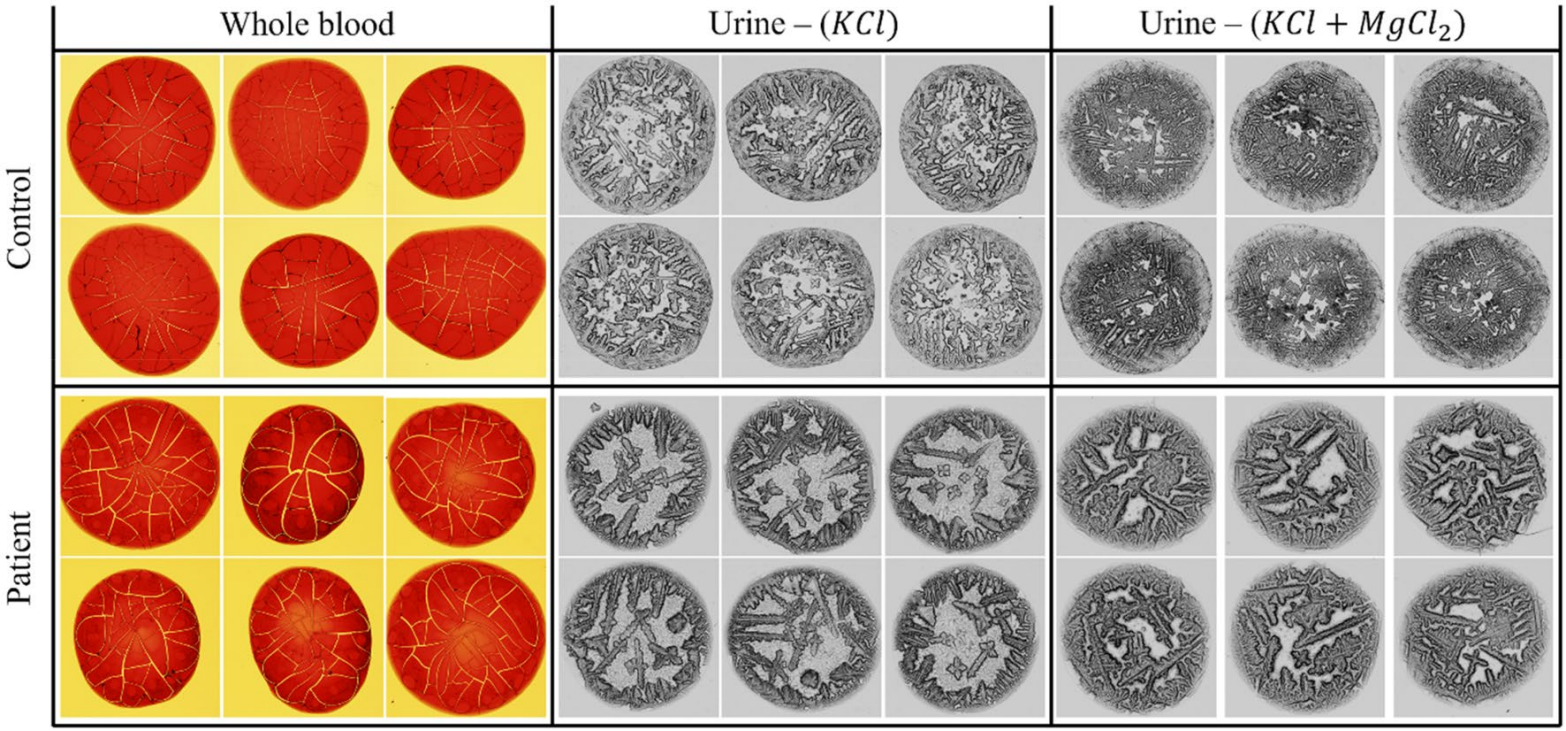
Examples of images of whole blood and urine droplet patterns from control individuals and bladder cancer patients. KCl, potassium chloride; MgCl_2_, magnesium chloride.

Whole blood samples were collected from BCa patients and control individuals in EDTA tubes before the surgical procedures, and samples were frozen and kept more than 2 h in − 80 °C freezers. Possible effects of freeze–thaw cycles were documented (Supplementary Fig. S1). Total hemolysis was achieved after three or more cycles (Supplementary Fig. S2). It was observed that droplet patterns (shadows, cracks, patterns, crystals, etc.) became consistent after this treatment. Droplet patterns were obtained following deposition of 2 µl blood on clear glass microscopy slides and drying droplets at room temperature. Images were taken under a light microscope (Fig. 1). 4–6 droplet images were taken for each case, and a total of 775 and 371 images were captured from patient samples and controls, respectively. Subsequently, machine learning and AI analyses were performed on these image collections.

### Imaging of urine droplets

First morning urine samples were collected from patients or controls and frozen in − 80 °C freezers. Urine samples were mixed 1:1 (volume:volume) with either KCl (1 M) or a KCl (1 M) and MgCl_2_ (1 M) mixture. Droplet patterns were obtained following deposition of 1 µl urine-salt mixture on clear glass microscopy slides and drying droplets at room temperature. Images were taken under a light microscope (Fig. 1). 4–6 droplet images were taken for each case. A total of 779 and 214 images were captured from the KCl mixed urine solutions of patients and controls, respectively. A total of 772 and 215 images were taken from KCl + MgCl_2_ mixed urine solutions of patients and controls, respectively. Machine learning and AI analyses were performed on these image collections.

### Feature extraction and CNN classification

**Figure 2:**
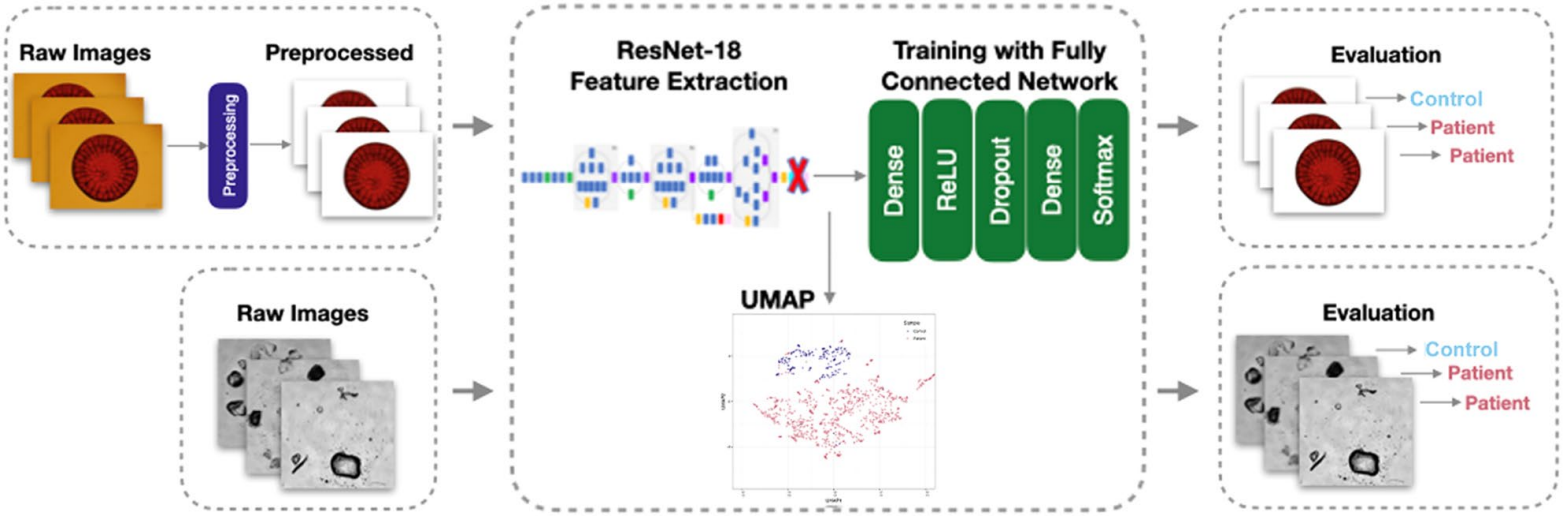
Schematic overview of the AI-based workflow for BCa patient/control classification on blood and urine samples.

In the literature, a common approach of designing a classification network, especially when limited image data are available, is to use a pretrained network in the first layers and add customized fully connected layers to the end. These pretrained network layers are known to be quite effective to extract distinguishing image features, which can be used for various computer vision tasks. The subsequent fully connected layers are task-specific, and their weights should be learned on the training set defined for the task at hand. In this study, we followed a similar approach (Fig. 2). In each CNN model, we used the ResNet-18 network architecture, pretrained on the ImageNet dataset without seeing any blood or urine droplet images^27^. Then, we trained the subsequent fully connected layers on the corresponding training set of blood and urine samples.

**Figure 3:**
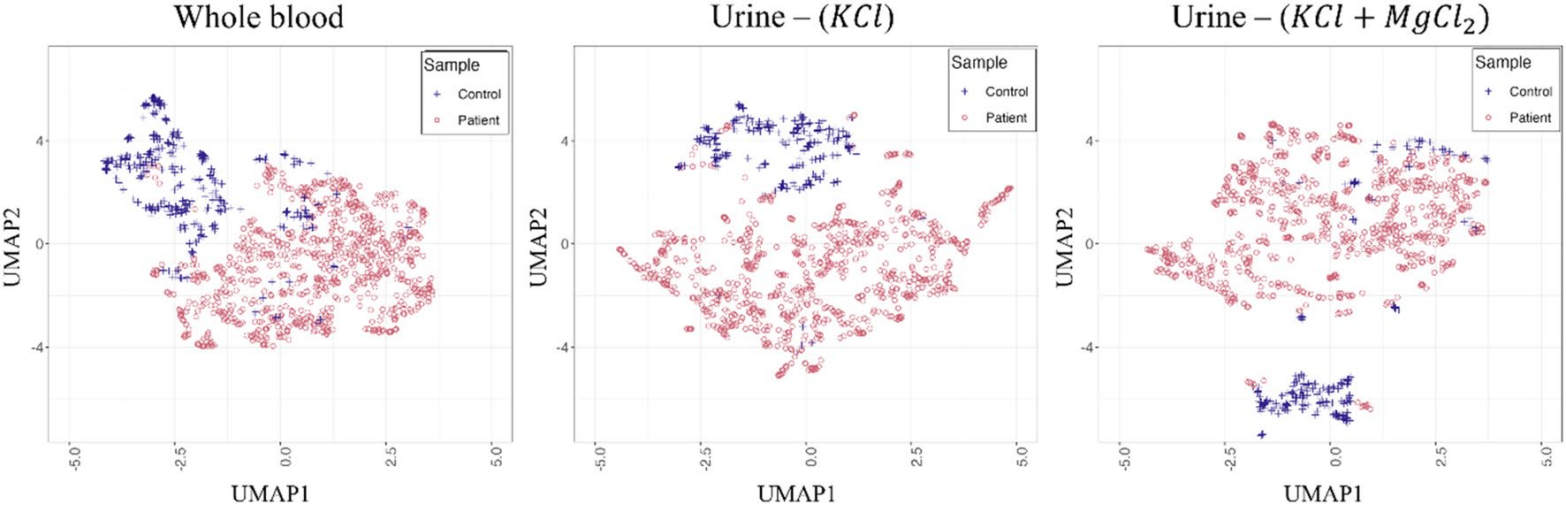
Distributions of the features extracted by the pretrained ResNet-18 network layers for the blood and urine droplets. Since these features were high-dimensional, the uniform manifold approximation and projection, or UMAP, was used for two-dimensional visualization. In these figures, blue and red dots represent the features extracted for the control and patient samples, respectively.

We first analyzed the effectiveness of features extracted by the pretrained ResNet-18 network in differentiating the patient-derived droplets and the control samples. To this end, the outputs (feature maps) of the last ResNet-18 layer were visualized. Since these feature maps were high-dimensional, we applied a nonlinear dimensionality reduction technique, namely uniform manifold approximation and projection or UMAP, which allows projecting a high-dimensional feature space into a two-dimensional space. The UMAP plots of the blood and urine samples are presented in Fig. 3. These plots revealed that the image-based patterns of droplet samples clustered together within the same class, which would enable accurate classification of the droplet images.

On the top of these pretrained layers, we separately trained the fully connected layers of three CNN classifiers: one on the set of blood samples and the other two on the sets of urine samples prepared adding two different salt solutions^28^. Each CNN was trained to classify a given unlabeled sample into two categories, as either “bladder cancer” or “not bladder cancer”. For classifier evaluation (testing part), the five-fold cross-validation technique was used due to the risk of overfitting. In this technique, the entire dataset of blood and urine samples was randomly divided into five folds and the testing part was repeated five times. In each trial, four folds (80% of the samples) were used to learn the network weights (of the fully connected layers) in the training and the remaining fold (20% of the samples), which was not used in the training at all, was used as the test set to calculate the performance metrics. At the end, the average metrics were calculated on the test sets of the five different trials. Note that in this technique, each fold will be used as the test set exactly once as an unseen throughout the learning.

**Figure 4:**
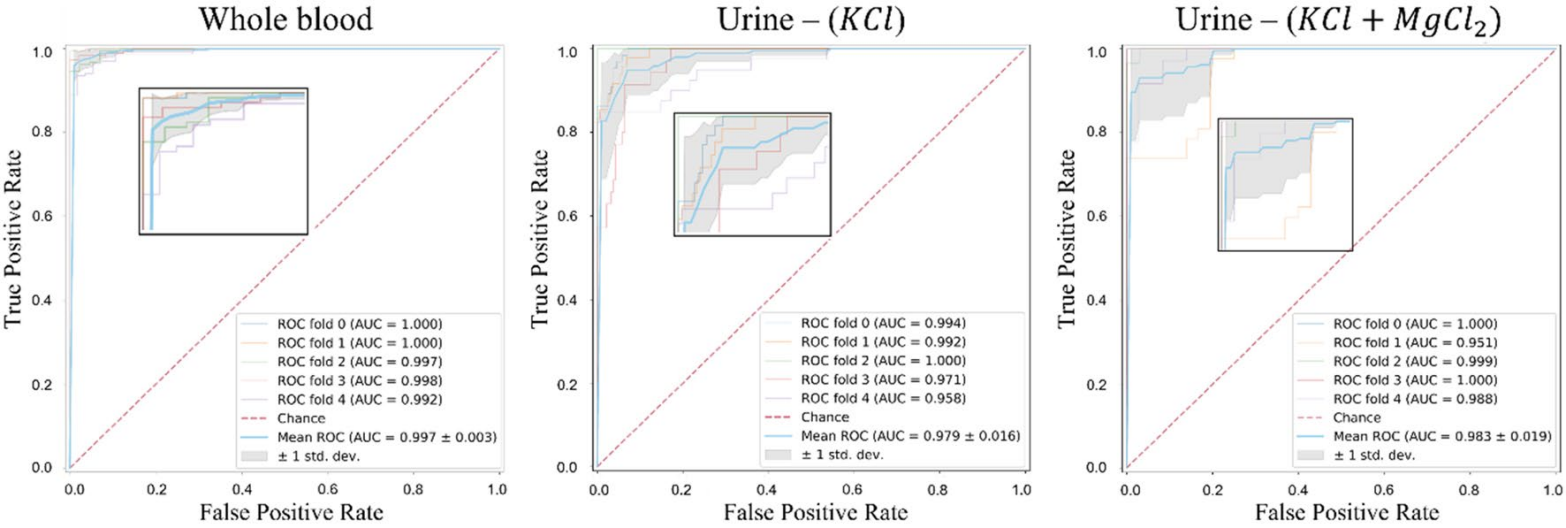
Receiver operating characteristic (ROC) curves for each of the five test folds. The areas under these curves (AUC) are separately reported for each fold together with their average.

The receiver operating characteristic (ROC) curves obtained for each of the five test set folds together with the area under these curves (AUC) were shown in Fig. 4. This figure demonstrated that our proposed model precisely differentiated the droplet images of cancerous patients and the control group with high AUCs. Table 2 also reported the sensitivity, specificity, and accuracy, separately for the blood and urine droplet samples. This table also revealed that the BCa and control groups were successfully classified for the blood samples, leading to high AUC (0.997 ± 0.003), accuracy (0.973 ± 0.016), sensitivity (0.977 ± 0.039), and specificity (0.972 ± 0.014). For the urine samples prepared using the KCl solution, the networks also led to high AUC (0.908 ± 0.066), accuracy (0.953 ± 0.034), sensitivity (0.987 ± 0.119), and specificity (0.829 ± 0.018). Likewise, the urine samples prepared using the KCl + MgCl_2_ solution were also differentiated with high AUC (0.988 ± 0.021), accuracy (0.748 ± 0.171), sensitivity (0.683 ± 0.386), and specificity (0.882 ± 0.171). We then provided the confusion matrices in Table 3 for the classification of whole blood, urine (KCl), and urine (KCl + MgCl2) samples together with the class-based classification accuracies. These confusion matrices were obtained by first finding the numbers on each test fold separately and then accumulating these numbers. Thus, they reflected the test performance. Additionally, in Table 3, we reported the class-based accuracies calculated on these accumulated numbers. These confusion matrices and class-based accuracies were consistent with the sensitivity and specificity metrics reported in Table 2.

**Table 2:** Average performance metrics obtained on the test folds together with their standard deviations.

	Whole blood	Urine—(KCl)	Urine—(KCl + MgCl2)
AUC	0.997 ± 0.003 (1.000, 1.000, 0.997, 0.998, 0.992)	0.979 ± 0.016 (0.994, 0.992, 1.000, 0.971, 0.958)	0.983 ± 0.019 (1.000, 0.951, 0.999, 1.000, 0.988)
Accuracy	0.973 ± 0.016 (0.996, 0.974, 0.961, 0.979, 0.956)	0.953 ± 0.034 (0.928, 0.955, 1.000, 0.914, 0.968)	0.748 ± 0.171 (0.739, 0.808, 0.635, 1.000, 0.556)
Sensitivity	0.977 ± 0.039 (1.000, 1.000, 0.977, 1.000, 0.911)	0.987 ± 0.119 (0.754, 0.854, 1.000, 0.686, 0.850)	0.683 ± 0.386 (1.000, 0.833, 0.470, 1.000, 0.111)
Specificity	0.972 ± 0.014 (0.994, 0.965, 0.957, 0.973, 0.971)	0.829 ± 0.018 (1.000, 0.983, 1.000, 0.957, 0.994)	0.882 ± 0.171 (0.625, 0.786, 1.000, 1.000, 1.000)

Metrics obtained on each of the five test folds are reported separately in parentheses.

*AUC* area under the receiver operating characteristic curve, *KCl* potassium chloride, *MgCl* magnesium chloride.

**Table 3:** Confusion matrices obtained by first finding the numbers on each test fold separately and then accumulating these numbers.

		Predicted		Class-based accuracy
		Control	Patient	
Whole blood				
Actual	Control	62	2	0.969
	Patient	3	127	0.977
Urine—(KCl)				
Actual	Control	53	11	0.828
	Patient	2	128	0.985
Urine—(KCl + MgCl_2_)				
Actual	Control	56	8	0.875
	Patient	41	89	0.685

Class-based accuracies were calculated on the accumulated numbers.

**Figure 5:**
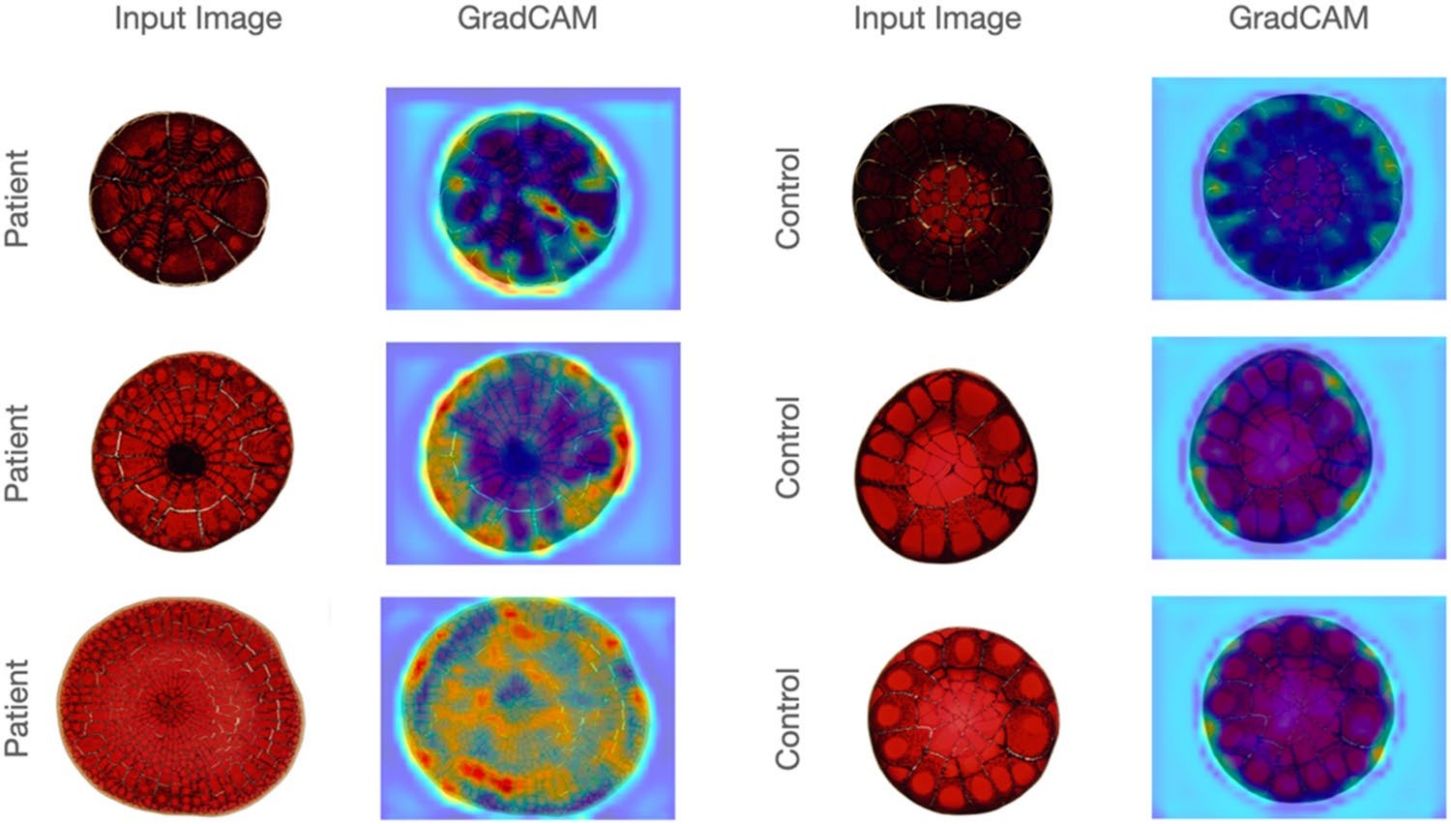
Maps of the highlighted specific areas that influenced the classification outcome for the exemplary blood samples from the patient and control groups. In these maps, warmer colors indicated more prominent regions used by the classifier. These maps were generated by the GradCam tool^29^.

We conducted an additional experiment using GradCam to get insights into the model’s decision-making process^29^. For the exemplary blood samples from the patient and control groups, the maps generated by GradCam were showed in Fig. 5. These maps included the highlighted specific areas that influenced the classification outcome, enhancing the interpretability of our classification network’s predictions. In these maps, warmer colors indicated more prominent regions used by the classifier. As shown in Fig. 5, the proposed model focused on both external and internal regions in the samples of the patient group whereas it produced weak signals internally and stronger signals externally in the samples of the control group. Note that we did not observe similar behavior for the urine samples.

**Figure 6:**
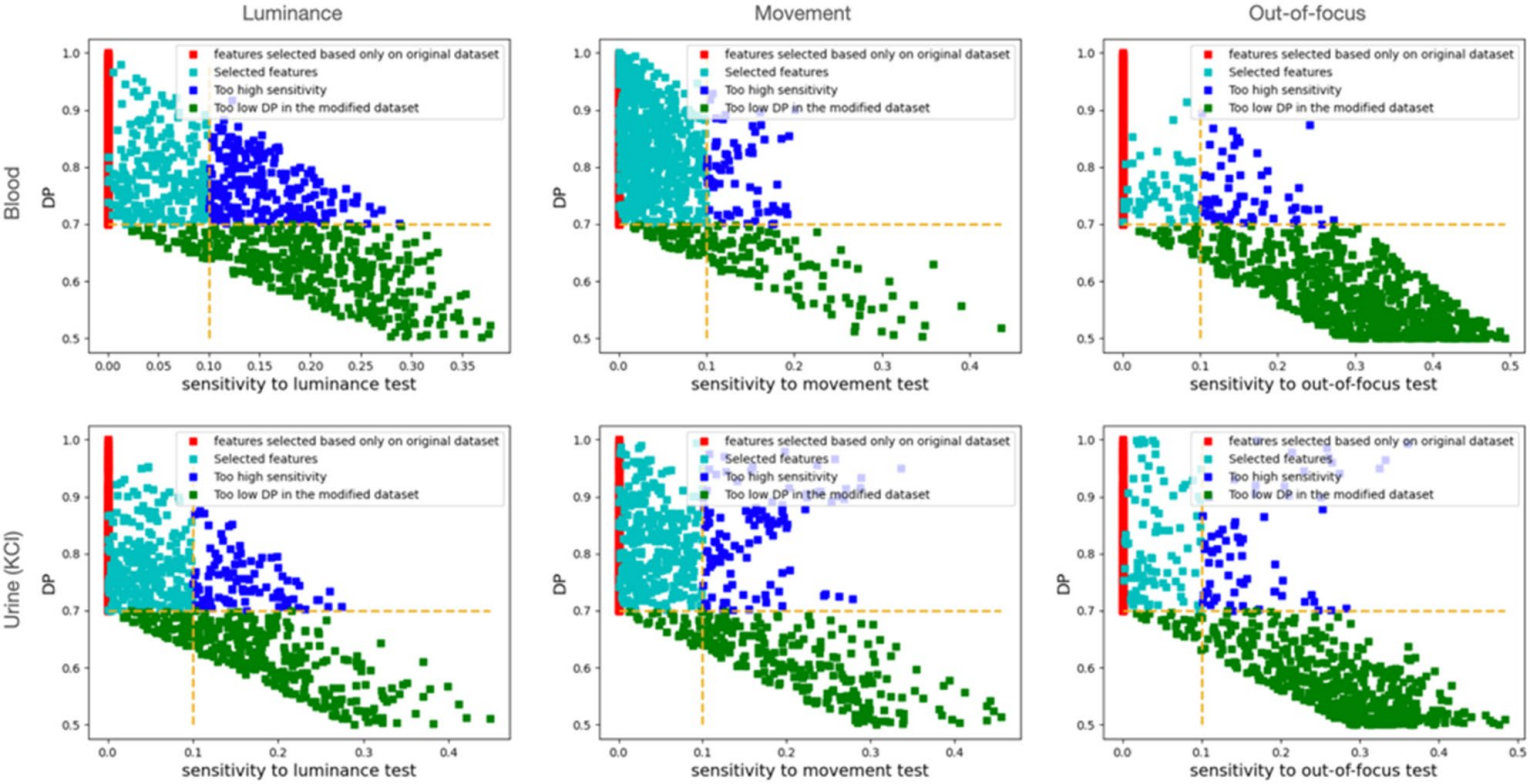
Distribution of the features with respect to their DP and the sensitivity to luminance, movement, and out-of-focus alterations for the blood and urine (KCl) droplet samples. These plots were generated by the Deep Manager tool^30^.

We also evaluated the quality of the extracted features with respect to environmental alterations not linked to the biological phenomena using the Deep-Manager tool^30^. The distribution of the features with respect to their DP and the sensitivity to luminance, movement, and out-of-focus alterations for the blood and urine (KCl) droplet samples were shown. In Fig. 6 we demonstrated that even with these alterations, there still existed a subset of features that showed less than 0.1 sensitivity to these alterations and led to DPs greater than 0.70, which was the minimum DP for the features selected based on the original dataset without any alterations. They led to slightly worse accuracy results compared to using the original feature set; 0.911 ± 0.076 for whole blood, 0.933 ± 0.029 for urine (KCI), and 0.711 ± 0.057 for urine (KCl + MgCl2) samples.

## Discussion

Bladder cancer is one of the most common urinary tract malignancies. It necessitates costly and invasive diagnostic and treatment methods as well as strict follow-up throughout patients' lifetime. For instance, cystoscopy, a commonly used diagnostic method for BCa, is an effective but invasive approach that requires qualified professionals and facilities for accurate diagnosis of the cancer^31–34^. Indeed, false negatives and procedure-related complications are not uncommon^35–37^. On the other hand, there are no specific and reliable serum or urine markers for BCa, rendering large screens and field diagnosis difficult tasks. Hence, practical, cost-effective, and accurate diagnostic tests need to be developed.

AI-based applications are widely used in modern diagnostic medicine, especially in the fields of radiology and pathology. Magnetic resonance imaging (MRI) scans, computed tomography (CT) results, microscopy images of stained tissue slides, and cytology analyses were among the primary sources of data used in AI-based applications^20^. Images of serum, or urine droplet patterns, have not so far been analyzed in the context of BCa^30^.

Several studies focused on the physical–chemical properties of evaporation and the consequences/effects on the formation of various droplet patterns and their reproducibility^38^. The behavior of droplet patterns is typical in pure liquids but has been found to be more complex in liquids containing multiple components^39^. We had previously analyzed the effects of salt mixtures on droplet pattern formation of bovine serum albumin (BSA) solutions and discovered that mixtures induced formation of various complex patterns^26^. Mimicking biological fluids using salt or isolated proteins (like BSA-salt solutions) deciphered how different patterns were forming and how specific they were^26^. In more complex contexts, evaporating liquids will turn into solid or gel, and these types of drops generally end up cracking and forming various patterns and morphologies^40,41^. Further analysis using organic solutions or original biological fluids such as blood and urine in their crude forms or in combination with other chemicals or solutions resulted in the formation of a variety of patterns, suggesting that patterns from diseased individuals may differ compared to those from healthy subjects^42^. Indeed, characteristics of blood plasma patterns was different in healthy individuals compared to hepatitis B positive patients^43^. In addition, analysis of anemic patients' dried whole blood patterns resulted in divergent pattern profiles compared to healthy individuals^44^. Moreover, morphological features of dried blood serum drops from patients with cancer, including breast and lung cancer, showed considerable differences^45^. In another study, dried human plasma patterns were used for metastatic carcinoma diagnosis^42^. However, the use of whole blood patterns for medical diagnosis was rarely reported^46^. Here, we used patterns formed by whole blood droplets for BCa diagnosis. In the case of urinary tract diseases, urine reflects changes in kidney and bladder biology, and it was used as another bodily fluid for BCa diagnosis.

In our study, an AI-based analysis method was developed using whole blood and urine samples and predicted BCa with high accuracy, sensitivity and specificity (Table 2). The proposed AI-based approach presented a number of advantages for BCa diagnosis. The use of whole blood and urine samples allows for rapid and reliable sample preparation and limited sample-to-sample variations. It should also be noted that after initial cycles, freeze–thaw cycles did not introduce sample instability and did not affect the results. The AI-based method of evaluation introduced standardization and automation of the result interpretation stage, and eliminated user-related bias. Hence, our approach has the potential to be developed as a rapid and practical BCa diagnosis test.

Convolutional neural networks (CNNs) are architecturally designed to handle spatially correlated data such as images^21,47^. Since AI models have the potential of alleviating many human errors arising from various factors, such as visual and mental fatigue, stress, and burn-out, their use as an assisted tool may prove beneficial to increase correct diagnosis and follow-up^20^. Transfer learning is another widely used strategy to combat overfitting especially when the dataset size is smaller than desired^48^. Our proposed CNN architecture, which was pretrained on the ImageNet dataset containing millions of images, can be systematically applied across blood and urine droplet images. This systematic application enables comparisons to reveal shared spatial behaviors and underlying morphological features that can precisely differentiate the image patterns specific to cancerous and control samples after partially training last layers with the target sample set. As also seen in their ROC curves (Fig. 3), these CNN-based models resulted in > 95% AUC for the BCa prediction on the images of whole blood and urine samples. Moreover, these models led to 0.977 sensitivity, 0.972 specificity, and 0.973 accuracy values for the blood samples, and 0.987 sensitivity, 0.829 specificity, and 0.953 accuracy values for the urine (KCl) samples (Table 2). This indicated the potential use of our proposed model as a candidate clinical assisted tool for BCa diagnosis on blood and urine samples.

In conclusion, the proposed AI-based method based on the analysis of blood and urine droplets presented herein may serve as a novel diagnosis and follow-up approach for BCa. Our CNN models, with the ResNet-18 network architecture pretrained on the ImageNet dataset, were used to classify these droplets taken from BCa patients and control individuals as either cancerous or non-cancerous with accuracies of 0.973 and 0.953 for the blood and urine (KCl) samples, respectively. These results, using a cohort of patients and controls, are very promising and indicate that AI-based models and methods might be used as non-invasive and accurate screening tests for the diagnosis of bladder cancer.

## Material and methods

### Collection of whole blood and urine samples

Study included 130 BCa patients admitted to the Urology Department of Marmara University Pendik Training and Research Hospital between 2018 and 2020. The control group was composed of 64 volunteers who had no BCa diagnosis in their lifetime. After informed consent, the blood and urine samples were taken from BCa patients before surgery. Blood and urine samples from patients or control subjects were collected in EDTA containing tubes and sterile urine containers (first urine sample of the morning), respectively, and stored in − 80 °C freezers until usage.

### Preparation of whole blood and urine droplets

The droplet formations were performed with or without solutions composed of salt mixtures (two mixtures, one obtained with adding 1 M KCl and the other one with 1 M KCl plus 1 M MgCl_2_). Salts were dissolved in deionized water as a stock solution (final concentration: 1 molar). Solution composition selection and optimization steps were previously described^26^. Urine samples were mixed with salt solutions at a 1:1 (volume:volume) ratio. 1 µl urine-salt mixtures or 2 µl blood droplets were deposited on clear glass microscopy slides (Sail Brand, cat. no. 7101) and left to dry at room temperature (22–24 °C). Six droplets per patient and control samples were prepared and imaged under the light microscope (Olympus BX53). Dried blood and urine droplets were imaged in adjusted optimum focus and pixel shifts (at 1360 × 1024 and 4140 × 4096 pixel resolution, respectively) for in-depth AI-based analysis. These deposited drops were all imaged in the RGB (Red, Green, and Blue) color space as well as in grayscale. Images were saved as TIFF files.

### Investigating the effects of freeze–thaw cycle

Freeze–thaw testing was conducted by exposing a whole blood sample to a freezing temperature (− 80 °C) for 24 h. Then, samples were thawed at room temperature and analyzed for possible changes by use of a hemocytometer under an inverted microscope. The cycles were repeated at least four times and dried droplet patterns were also documented as microscope images.

### Investigation of droplet images by AI

Due to its widespread use and success in machine learning and image analysis, a deep neural network, a ResNet-18 network pre-trained on the ImageNet dataset, was systematically applied across the collected whole blood and urine droplet images. This enables comparisons to reveal shared spatial behaviors and underlying morphological patterns. Images of blood and urine samples were categorized into two main groups: “bladder cancer” and “not bladder cancer”. Preparation and processing of data was completed in two steps. First, data cleaning was applied to make the image data ready for AI-based analysis. In the second step, the data was preprocessed, models (networks) were trained, and the results were analyzed. Before training, the blood samples were preprocessed by background correction; no postprocessing was used for the urine samples.

### CNN architecture and training

We developed three CNN-based models for BCa patient/control classification, one using the blood droplet images, and the other two using the urine droplet images prepared adding two different salt mixtures^28^. Each model used the ResNet-18 network architecture with the modified last layers, which were one fully connected layer with 512 hidden units followed by rectified linear unit (ReLU) activation and dropout regularization and another fully connected layer with the softmax activation. The network parameters (weights) were learned using the transfer learning approach. To do so, the weights of the network’s first layers were taken from the ResNet-18 model pre-trained on the ImageNet dataset and the last fully connected layers were trained from scratch on full-size droplet images with the 1360 × 1024 and 4140 × 4096 pixel resolution for the classification of blood and urine samples, respectively. To prevent the loss of important spatial context within an image, image tiling was not preferred as using the entire image provides a more complete picture of the object or scene being analyzed.

The model was trained for the maximum of 512 epochs, where an early stopping method was used to stop training if there was no improvement on the performance of validation images over the last 20 consecutive epochs to achieve a better generalization with an unseen sample set. The batch size was selected as 64. The categorical cross-entropy was used as the loss function. Model parameters were optimized via the Adam optimizer with a learning rate of 2 × 10^−4^ and a 1 × 10^−5^ L2 weight decay. To mitigate the negative effect of having the class imbalance problem, the majority class (BCa patient samples) were under sampled during training to match the contribution of the losses defined on the images of the minority class (control samples).

### Statistical analysis

Statistical evaluation of the clinical data that may affect blood and urine samples obtained from BCa patient and control subjects were performed by IBM SPSS Statistics (Version 20).

### Ethical approval

This study was approved by the Ethics Committee of Marmara University School of Medicine (Protocol No: 09.2018.367). All procedures were carried out in accordance with the ethical rules and the principles of the Declaration of Helsinki. Confirms that informed consent was obtained from all participants.

### Supplementary Information

Supplementary Information.

## Data Availability

The datasets used and/or analyzed during the current study are available from the corresponding author on reasonable request.

## Abbreviations

CNN: Convolutional neural network
BCa: Bladder cancer
UCC: Urothelial cell carcinoma
KCl: Potassium chloride
MgCl_2_: Magnesium chloride
MIBC: Muscle-invasive bladder cancer
NMIBC: Non-muscle invasive bladder cancer
AI: Artificial intelligence
UMAP: Uniform manifold approximation and projection
AUC: Area under the curve
ROC curve: Receiver operating characteristic curve

## References

[1] Sung H (2021). Global cancer statistics 2020: GLOBOCAN estimates of incidence and mortality worldwide for 36 cancers in 185 countries. CA Cancer J. Clin..

[2] Antoni S (2017). Bladder cancer incidence and mortality: A global overview and recent trends. Eur. Urol..

[3] Silverman, D. T., Koutros, S., Figueroa, J. D., Prokunina-Olsson, L. & Rothman, N. in *Cancer Epidemiology and Prevention* (ed Michael Thun) 977–996 (Oxford Academic, 2017).

[4] Teoh JY (2020). Global trends of bladder cancer incidence and mortality, and their associations with tobacco use and gross domestic product per capita. Eur. Urol..

[5] Tran L, Xiao JF, Agarwal N, Duex JE, Theodorescu D (2021). Advances in bladder cancer biology and therapy. Nat. Rev. Cancer.

[6] Sanli O (2017). Bladder cancer. Nat. Rev. Dis. Primers.

[7] Berdik C (2017). Unlocking bladder cancer. Nature.

[8] Schiffer E (2009). Prediction of muscle-invasive bladder cancer using urinary proteomics. Clin. Cancer Res..

[9] Habuchi, T. in *Bladder Tumors: Cancer Drug Discovery and Development* (eds B. L. Lokeshwar, A. S. Merseburger, & S. H. Hautmann) 139–163 (Humana Press., 2011).

[10] McNeil, B. K., Ekwenna, O. O. & Getzenberg, R. H. in *Bladder Tumors: Cancer Drug Discovery and Development.* (eds V. Lokeshwar, A. Merseburger, & S. Hautmann) (Humana Press, 2011).

[11] Sorace J (2012). Integrating pathology and radiology disciplines: An emerging opportunity?. BMC Med..

[12] Xiao C, Choi E, Sun J (2018). Opportunities and challenges in developing deep learning models using electronic health records data: A systematic review. J. Am. Med. Inform. Assoc..

[13] Vijayan V, Connolly JP, Condell J, McKelvey N, Gardiner P (2021). Review of wearable devices and data collection considerations for connected health. Sensors.

[14] Karczewski KJ, Snyder MP (2018). Integrative omics for health and disease. Nat. Rev. Genet..

[15] Kumar Y, Koul A, Singla R, Ijaz MF (2023). Artificial intelligence in disease diagnosis: A systematic literature review, synthesizing framework and future research agenda. J. Amb. Intell. Hum. Comput..

[16] Goldenberg SL, Nir G, Salcudean SE (2019). A new era: Artificial intelligence and machine learning in prostate cancer. Nat. Rev. Urol..

[17] Capek D (2023). EmbryoNet: Using deep learning to link embryonic phenotypes to signaling pathways. Nat. Methods.

[18] D'Orazio M (2020). Deciphering cancer cell behavior from motility and shape features: Peer prediction and dynamic selection to support cancer diagnosis and therapy. Front. Oncol..

[19] D'Orazio M (2022). Machine learning phenomics (MLP) combining deep learning with time-lapse-microscopy for monitoring colorectal adenocarcinoma cells gene expression and drug-response. Sci. Rep..

[20] Borhani S, Borhani R, Kajdacsy-Balla A (2022). Artificial intelligence: A promising frontier in bladder cancer diagnosis and outcome prediction. Crit. Rev. Oncol. Hematol..

[21] Goodfellow I (2020). Generative adversarial networks. Commun. ACM.

[22] Chen R, Zhang L, Zang D, Shen W (2016). Blood drop patterns: Formation and applications. Adv. Colloid Interface Sci..

[23] Sobac B, Brutin D (2014). Desiccation of a sessile drop of blood: Cracks, folds formation and delamination. Colloids Surf. A Physicochem. Eng. Asp..

[24] Lee CS, Yoon CY, Witjes JA (2008). The past, present and future of cystoscopy: The fusion of cystoscopy and novel imaging technology. BJU Int..

[25] Marieb EN, Keller SN (2017). Essentials of Human Anatomy & Physiology.

[26] Pathak B, Christy J, Sefiane K, Gozuacik D (2020). Complex pattern formation in solutions of protein and mixed salts using dehydrating sessile droplets. Langmuir.

[27] He K, Zhang X, Ren S, Sun J (2016). Deep residual learning for image recognition. Proc. IEEE Conf. Comput. Vis. Pattern Recognit..

[28] Noorbakhsh J (2020). Deep learning-based cross-classifications reveal conserved spatial behaviors within tumor histological images. Nat. Commun..

[29] Selvaraju RR (2017). Grad-cam: Visual explanations from deep networks via gradient-based localization. Proc. IEEE Conf. Comput. Vis. Pattern Recognit..

[30] Mencattini A (2023). Deep-Manager: A versatile tool for optimal feature selection in live-cell imaging analysis. Commun. Biol..

[31] Biardeau X, Lam O, Ba V, Campeau L, Corcos J (2017). Prospective evaluation of anxiety, pain, and embarrassment associated with cystoscopy and urodynamic testing in clinical practice. Can Urol. Assoc. J..

[32] Chang SS (2016). Diagnosis and treatment of non-muscle invasive bladder cancer: AUA/SUO guideline. J. Urol..

[33] Faiena I, Rosser CJ, Chamie K, Furuya H (2019). Diagnostic biomarkers in non-muscle invasive bladder cancer. World J. Urol..

[34] Oeyen E (2019). Bladder cancer diagnosis and follow-up: The current status and possible role of extracellular vesicles. Int. J. Mol. Sci..

[35] Burke DM, Shackley DC, O'Reilly PH (2002). The community-based morbidity of flexible cystoscopy. BJU Int..

[36] Herr HW, Donat SM (2008). Quality control in transurethral resection of bladder tumours. BJU Int..

[37] Raitanen MP (2001). Routine follow-up cystoscopy in detection of recurrence in patients being monitored for bladder cancer. Ann. Chir. Gynaecol..

[38] Cameron JM, Butler HJ, Palmer DS, Baker MJ (2018). Biofluid spectroscopic disease diagnostics: A review on the processes and spectral impact of drying. J Biophotonics.

[39] Diddens C (2017). Evaporating pure, binary and ternary droplets: Thermal effects and axial symmetry breaking. J. Fluid Mech..

[40] Annarelli C, Fornazero J, Bert J, Colombani J (2001). Crack patterns in drying protein solution drops. Eur. Phys. J. E.

[41] Pearce EI, Tomlinson A (2000). Spatial location studies on the chemical composition of human tear ferns. Ophthalmic Physiol. Opt..

[42] Rapis E (2002). A change in the physical state of a nonequilibrium blood plasma protein film in patients with carcinoma. Tech. Phys..

[43] Martusevich AK, Zimin Y, Bochkareva A (2007). Morphology of dried blood serum specimens of viral hepatitis. Hepatitis Monthly.

[44] Brutin D, Sobac B, Loquet B, Sampol J (2011). Pattern formation in drying drops of blood. J. Fluid Mech..

[45] Yakhno TA (2005). The informative-capacity phenomenon of drying drops. IEEE Eng. Med. Biol. Mag..

[46] Brutin D, Sobac B, Nicloux C (2012). Influence of substrate nature on the evaporation of a sessile drop of blood. J. Heat Transfer..

[47] Bakator M, Radosav D (2018). Deep learning and medical diagnosis: A review of literature. Multimod. Technol. Interact..

[48] Weiss K, Khoshgoftaar TM, Wang D (2016). A comprehensive survey on transfer learning. J. Big Data.

