# Supplementary material for: Artificial intelligence assisted patient blood and urine droplet pattern analysis for non-invasive and accurate diagnosis of bladder cancer
Source: Sci Rep. 2024 Jan 30;14:2488. doi: 10.1038/s41598-024-52728-7 (PMC10827787; doi:10.1038/s41598-024-52728-7)
Supplement: Supplementary file 1 — Supplementary Information. [file 41598_2024_52728_MOESM1_ESM.docx]

**Supplementary Table S1**: List of control individuals considered in this study. C, control.

| **Control number** | **Gender** | **Age** |
| --- | --- | --- |
| C1 | Male | 63 |
| C2 | Male | 74 |
| C3 | Male | 66 |
| C4 | Female | 78 |
| C5 | Female | 62 |
| C6 | Male | 67 |
| C7 | Female | 70 |
| C8 | Male | 32 |
| C9 | Male | 49 |
| C10 | Male | 72 |
| C11 | Male | 30 |
| C12 | Male | 45 |
| C13 | Male | 52 |
| C14 | Male | 36 |
| C15 | Male | 51 |
| C16 | Male | 33 |
| C17 | Male | 46 |
| C18 | Male | 41 |
| C19 | Male | 39 |
| C20 | Female | 54 |
| C21 | Female | 54 |
| C22 | Female | 73 |
| C23 | Female | 28 |
| C24 | Male | 50 |
| C25 | Female | 27 |
| C26 | Male | 28 |
| C27 | Male | 61 |
| C28 | Female | 52 |
| C29 | Female | 53 |
| C30 | Female | 72 |
| C31 | Male | 47 |
| C32 | Male | 20 |
| C33 | Female | 44 |
| C34 | Female | 79 |
| C35 | Female | 39 |
| C36 | Female | 71 |
| C37 | Male | 58 |
| C38 | Male | 46 |
| C39 | Female | 40 |
| C40 | Male | 65 |
| C41 | Female | 32 |
| C42 | Male | 38 |
| C43 | Female | 39 |
| C44 | Female | 83 |
| C45 | Male | 60 |
| C46 | Male | 78 |
| C47 | Female | 37 |
| C48 | Male | 61 |
| C49 | Female | 56 |
| C50 | Female | 40 |
| C51 | Female | 31 |
| C52 | Female | 47 |
| C53 | Male | 54 |
| C54 | Male | 35 |
| C55 | Male | 43 |
| C56 | Male | 42 |
| C57 | Female | 73 |
| C58 | Female | 56 |
| C59 | Female | 74 |
| C60 | Female | 62 |
| C124P | Male | 76 |
| C135P | Male | 62 |
| C138P | Male | 70 |
| C141P | Male | 61 |

**Supplementary Table S2**: List of cancer patients considered in this study^.^ UCC, urothelial cell carcinoma; LG, low grade; HG, high grade; “-”, information not known; P, patients; Cis, carcinoma in situ; NMBIC, non-muscle invasive bladder cancer; MBIC, muscle invasive bladder cancer.

| **Patient number** | **Gender** | **Age** | **Type** | **Origin of tumor** | **Grade** | **Stage** | **Invasiveness** |
| --- | --- | --- | --- | --- | --- | --- | --- |
| P1 | Male | 72 | UCC | Primary | HG | pT1 | NMIBC |
| P2 | Male | 61 | UCC | Primary | HG | pT1 | NMIBC |
| P3 | Male | 59 | UCC | Primary | LG | pTa | NMIBC |
| P4 | Male | 54 | UCC | Primary | HG | pTa | NMIBC |
| P5 | Male | 72 | UCC | Primary | LG | pTa | NMIBC |
| P6 | Male | 53 | UCC | Recurrence | HG | pT1 | NMIBC |
| P7 | Female | 82 | UCC | Primary | HG | pT1 | NMIBC |
| P9 | Male | 62 | UCC | Primary | HG | pT2 | MIBC |
| P11 | Male | 62 | UCC | Primary | LG | pTa | NMIBC |
| P12 | Male | 80 | UCC | Primary | LG | pTa | NMIBC |
| P13 | Male | 59 | UCC | Primary | HG | pT2 | MIBC |
| P14 | Male | 65 | UCC | Primary | - | pT1 | NMIBC |
| P15 | Male | 77 | UCC | Primary | HG | pT1 | NMIBC |
| P17 | Female | 61 | UCC | Primary | HG | pT1 | NMIBC |
| P18 | Male | 49 | UCC | Primary | HG | pT2 | MIBC |
| P19 | Male | 80 | UCC | Recurrence | HG | pTa | NMIBC |
| P21 | Male | 69 | UCC | Recurrence | LG | pTa | NMIBC |
| P22 | Male | 73 | UCC | Recurrence | HG | pT1 | NMIBC |
| P23 | Male | 62 | UCC | Primary | LG | pTa | NMIBC |
| P24 | Male | 60 | UCC | Primary | HG | pT1 | NMIBC |
| P25 | Male | 69 | UCC | Primary | HG | pTa | NMIBC |
| P26 | Male | 39 | UCC | Primary | LG | pTa | NMIBC |
| P27 | Female | 66 | UCC | Primary | HG | pT2 | MIBC |
| P28 | Female | 66 | UCC | Primary | HG | pT1 | NMIBC |
| P29 | Male | 72 | UCC | Primary | HG | pT1 | NMIBC |
| P30 | Male | 23 | UCC | Primary | LG | pTa | NMIBC |
| P31 | Male | 68 | UCC | Primary | HG | pTa | NMIBC |
| P32 | Male | 70 | UCC | Primary | LG | pTa | NMIBC |
| P33 | Male | 49 | UCC | Recurrence | HG | pT1 | NMIBC |
| P34 | Female | 73 | UCC | Primary | HG | pT1 | NMIBC |
| P35 | Male | 65 | UCC | Primary | LG | pTa | NMIBC |
| P36 | Male | 70 | UCC | Primary | HG | pT1 | NMIBC |
| P37 | Male | 52 | UCC | Primary | LG | pTa | NMIBC |
| P38 | Male | 86 | UCC | Primary | HG | pT2 | MIBC |
| P39 | Male | 74 | UCC | Primary | HG | pT1 | NMIBC |
| P40 | Male | 51 | UCC | Recurrence | LG | pTa | NMIBC |
| P41 | Male | 50 | UCC | Primary | HG | pTa | NMIBC |
| P42 | Male | 69 | UCC | Primary | HG | pT1 | NMIBC |
| P43 | Male | 74 | UCC | Primary | HG | pT2 | MIBC |
| P44 | Male | 59 | UCC | Recurrence | HG | pT1 | NMIBC |
| P45 | Female | 89 | UCC | Primary | HG | pT1 | NMIBC |
| P46 | Male | 67 | UCC | Primary | LG | pTa | NMIBC |
| P47 | Male | 63 | UCC | Primary | LG | pTa | NMIBC |
| P48 | Male | 51 | UCC | Primary | HG | pT2 | MIBC |
| P50 | Male | 76 | UCC | Primary | LG | pTa | NMIBC |
| P52 | Male | 84 | UCC | Primary | HG | pT1 | NMIBC |
| P53 | Male | 46 | UCC | Primary | HG | pTa | NMIBC |
| P54 | Female | 70 | UCC | Primary | LG | pTa | NMIBC |
| P55 | Male | 48 | UCC | Primary | LG | pT1 | NMIBC |
| P56 | Male | 71 | UCC | Primary | LG | pTa | NMIBC |
| P57 | Male | 69 | UCC | Primary | LG | pTa | NMIBC |
| P59 | Male | 69 | UCC | Primary | HG | pT1 | NMIBC |
| P60 | Male | 71 | UCC | Primary | HG | pT1 | NMIBC |
| P61 | Male | 61 | UCC | Primary | HG | pT1 | NMIBC |
| P62 | Male | 68 | UCC | Primary | LG | pT1 | NMIBC |
| P63 | Male | 67 | UCC | Primary | HG | pT2 | MIBC |
| P64 | Male | 78 | UCC | Primary | HG | pTa | NMIBC |
| P65 | Male | 82 | UCC | Primary | LG | pT1 | NMIBC |
| P66 | Male | 73 | UCC | Primary | HG | pTa | NMIBC |
| P67 | Male | 69 | UCC | Primary | HG | pTa | NMIBC |
| P68 | Male | 45 | UCC | Primary | LG | pTa | NMIBC |
| P69 | Male | 72 | UCC | Recurrence | HG | pT1 | NMIBC |
| P70 | Male | 79 | UCC | Recurrence | HG | pT1 | NMIBC |
| P71 | Female | 65 | UCC | Primary | LG | pTa | NMIBC |
| P73 | Male | 63 | UCC | Recurrence | LG | pTa | NMIBC |
| P74 | Male | 66 | UCC | Recurrence | LG | pTa | NMIBC |
| P75 | Male | 75 | UCC | Primary | HG | pT1 | NMIBC |
| P76 | Male | 64 | UCC | Primary | HG | pT1 | NMIBC |
| P78 | Male | 71 | UCC | Primary | HG | pT1 | NMIBC |
| P79 | Male | 68 | UCC | Recurrence | LG | pTa | NMIBC |
| P80 | Female | 64 | UCC | Primary | HG | pT1 | NMIBC |
| P81 | Male | 36 | UCC | Primary | LG | pTa | NMIBC |
| P82 | Male | 45 | UCC | Primary | LG | pTa | NMIBC |
| P83 | Male | 68 | UCC | Primary | HG | pT1 | NMIBC |
| P84 | Male | 52 | UCC | Recurrence | HG | pTa | NMIBC |
| P85 | Female | 83 | UCC | Recurrence | HG | pT2 | MIBC |
| P86 | Female | 62 | UCC | Recurrence | LG | pTa | NMIBC |
| P87 | Male | 83 | UCC | Recurrence | LG | pTa | NMIBC |
| P88 | Male | 58 | UCC | Recurrence | LG | pTa | NMIBC |
| P89 | Female | 40 | UCC | Recurrence | LG | pTa | NMIBC |
| P91 | Male | 59 | UCC | Recurrence | LG | pTa | NMIBC |
| P92 | Male | 73 | UCC | Primary | LG | pTa | NMIBC |
| P95 | Female | 55 | UCC | Primary | HG | pT1 | NMIBC |
| P96 | Male | 86 | UCC | Primary | HG | pT1 | NMIBC |
| P97 | Male | 79 | UCC | Primary | HG | pT2 | MIBC |
| P98 | Male | 61 | UCC | Primary | LG | pTa | NMIBC |
| P99 | Male | 61 | UCC | Primary | HG | pT1 | NMIBC |
| P100 | Male | 49 | UCC | Recurrence | HG | pT1 | NMIBC |
| P101 | Male | 81 | UCC | Primary | LG | pTa | NMIBC |
| P103 | Male | 67 | UCC | Recurrence | LG | pTa | NMIBC |
| P104 | Male | 57 | UCC | Recurrence | LG | pTa | NMIBC |
| P105 | Female | 68 | UCC | Primary | HG | pT1 | NMIBC |
| P106 | Male | 59 | UCC | Primary | LG | pT1 | NMIBC |
| P107 | Male | 73 | UCC | Primary | LG | pTa | NMIBC |
| P108 | Male | 53 | UCC | Primary | LG | pTa | NMIBC |
| P109 | Female | 54 | UCC | Recurrence | LG | pTa | NMIBC |
| P110 | Male | 69 | UCC | Primary | LG | pTa | NMIBC |
| P111 | Male | 62 | UCC | Recurrence | LG | pTa | NMIBC |
| P112 | Male | 66 | UCC | Primary | LG | pTa | NMIBC |
| P113 | Male | 37 | UCC | Primary | HG | pT1 | NMIBC |
| P114 | Female | 62 | UCC | Primary | LG | pTa | NMIBC |
| P115 | Male | 68 | UCC | Primary | HG | pTa | NMIBC |
| P117 | Male | 67 | UCC | Primary | LG | pTa | NMIBC |
| P118 | Male | 74 | UCC | Primary | HG | pT2 | MIBC |
| P119 | Male | 63 | UCC | Primary | HG | pTa | NMIBC |
| P120 | Male | 88 | UCC | Recurrence | HG | pTa | NMIBC |
| P121 | Female | 73 | UCC | Recurrence | LG | pTa | NMIBC |
| P122 | Female | 61 | UCC | Primary | HG | pT1 | NMIBC |
| P123 | Female | 63 | UCC | Primary | LG | pTa | NMIBC |
| P125 | Male | 51 | UCC | Primary | LG | pT1 | NMIBC |
| P126 | Female | 72 | UCC | Recurrence | HG | pT1 | NMIBC |
| P127 | Male | 62 | UCC | Primary | HG | pT1 | NMIBC |
| P128 | Male | 60 | UCC | Primary | LG | Cis | NMIBC |
| P129 | Male | 56 | UCC | Primary | HG | pTa | NMIBC |
| P131 | Male | 66 | UCC | Primary | HG | pT1 | NMIBC |
| P132 | Male | 42 | UCC | Recurrence | LG | pTa | NMIBC |
| P134 | Male | 62 | UCC | Primary | HG | pT2 | MIBC |
| P136 | Male | 68 | UCC | Primary | HG | pT1 | NMIBC |
| P137 | Male | 74 | UCC | Recurrence | LG | pTa | NMIBC |
| P139 | Male | 54 | UCC | Recurrence | LG | pTa | NMIBC |
| P140 | Male | 59 | UCC | Recurrence | LG | pTa | NMIBC |
| P143 | Male | 63 | UCC | Recurrence | LG | pTa | NMIBC |
| P144 | Male | 61 | UCC | Primary | LG | pTa | NMIBC |
| P145 | Male | 73 | UCC | Recurrence | LG | pT1 | NMIBC |
| P146 | Male | 79 | UCC | Primary | HG | pT1 | NMIBC |
| P147 | Male | 67 | UCC | Primary | HG | pT1 | NMIBC |
| P148 | Male | 86 | UCC | Primary | LG | Cis | NMIBC |
| P149 | Male | 50 | UCC | Primary | LG | pTa | NMIBC |
| P150 | Male | 66 | UCC | Recurrence | HG | pTa | NMIBC |
| P151 | Male | 64 | UCC | Primary | LG | pT1 | NMIBC |


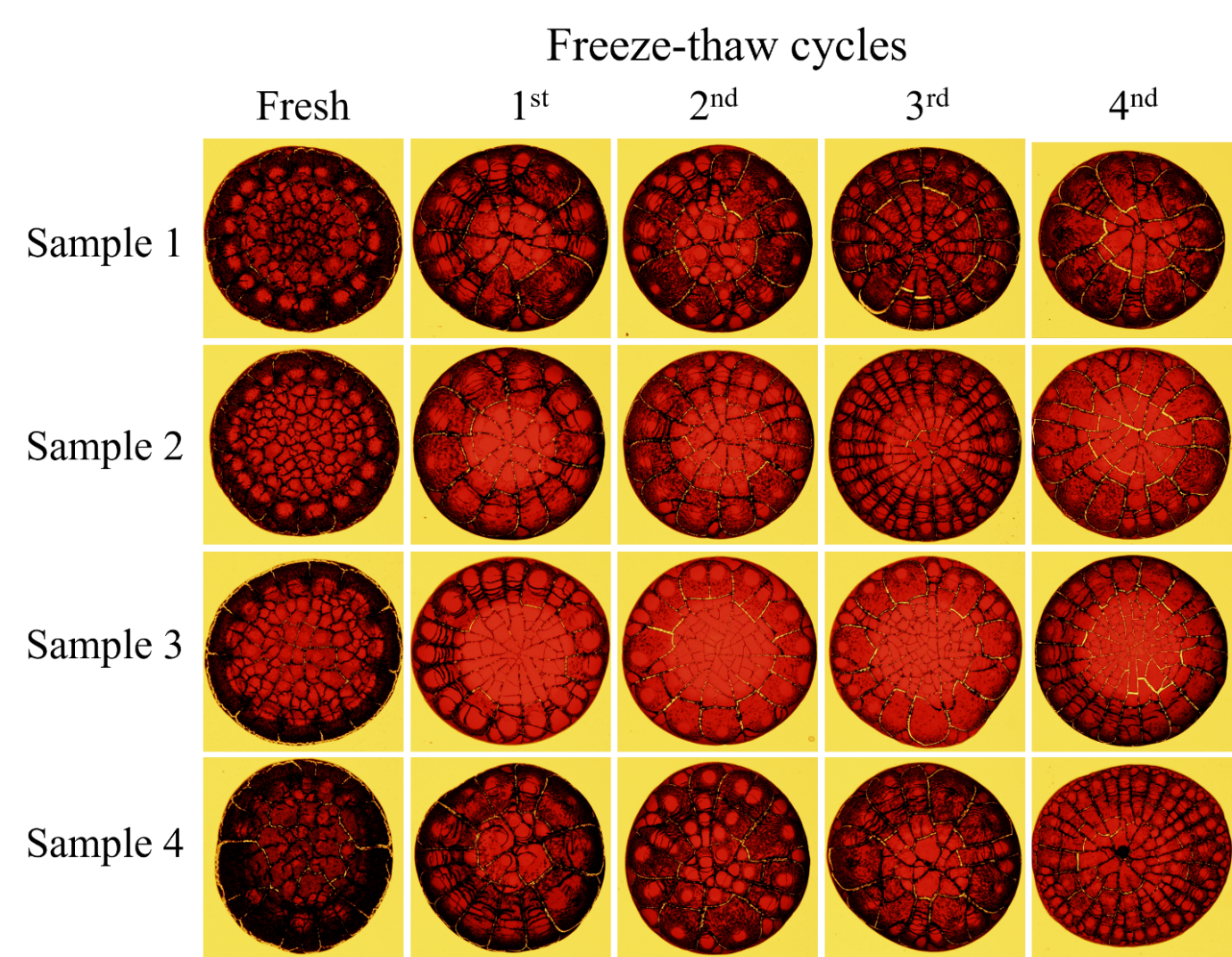


**Supplementary Figure S1**: Effects of freeze-thaw cycles on the pattern of whole blood droplets.


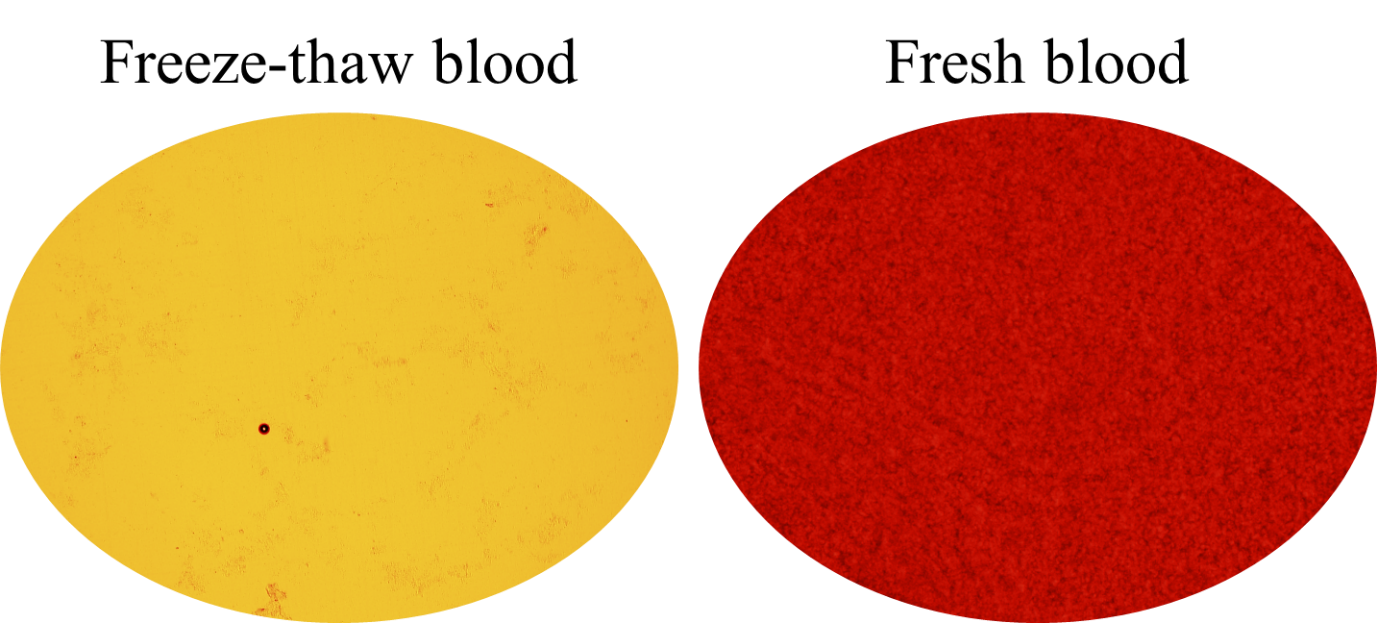


**Supplementary Figure S2**: Visualization of blood cells by hemocytometer.
